# An assessment of US dairy industry needs for digital tools in food safety and quality management

**DOI:** 10.3168/jdsc.2026-1012

**Published:** 2026-03-12

**Authors:** Jun Su, Martin Wiedmann

**Affiliations:** Department of Food Science, Cornell University, Ithaca, NY 14850

## Abstract

Digital tools can enhance food safety and quality management in the dairy industry, but their adoption remains uneven. To assess industry needs for digital tools that support food safety and quality management, 23 food safety and quality professionals from 18 US dairy companies, representing all major US regions, were interviewed through the National Science Foundation Regional I-Corps program. The participants reported using digital tools for compliance, environmental monitoring programs (EMP), and predictive modeling. High priority needs they reported included facilitation of EMP and clear demonstration of return on investment. Larger companies were more likely to adopt digital tools, whereas representatives from smaller firms reported relying on paper-based systems or spreadsheets. Overall, the findings highlight key opportunities for digital tool development and provide guidance for future research and implementation of digital tools in the dairy industry.

## Highlights

• Digital adoption varies; larger firms frequently use advanced tools, and smaller ones typically rely on paper.
• Regulatory rules push companies to adopt tools ensuring compliance and traceability.
• Tools are adopted when they show clear ROI.
• Centralized, connected data are needed to replace scattered information.

## Graphical Abstract

**Figure:**
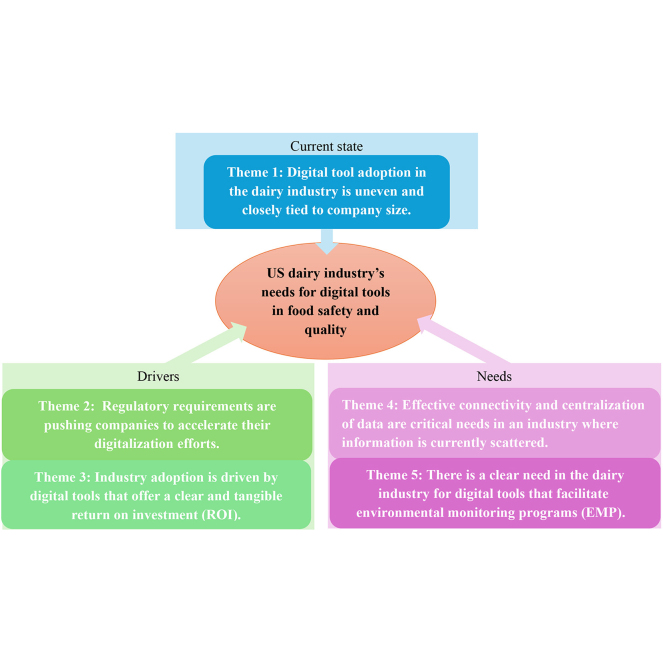
**Summary:**Adoption of digital tools for food safety and quality in the US dairy industry is uneven. Larger companies often have advanced digital systems and increasingly use tools for predictive insights and operational optimization, whereas smaller firms are reported to largely remain reliant on manual, paper-based processes. Key drivers include regulatory requirements and the need for clear return on investment (ROI). Critical needs include centralized, connected data systems to replace fragmented, manual workflows and support timely, accurate decision-making. There is also a growing demand for environmental monitoring program (EMP) software to map, track, and analyze environmental data, enabling proactive food safety management.

Food safety and quality management in the dairy sector have become increasingly complex, driven by evolving regulatory requirements, the growing diversity of dairy products, and heightened customer expectations for safety, transparency, and quality (Haldar et al., 2022; Hooda et al., 2025). These factors place considerable demands on food safety and quality professionals, who must ensure compliance, manage risks, and maintain operational efficiency in a rapidly changing environment (Manning and Baines, 2004). Digital tools, such as software for compliance tracking, predictive risk assessment, and data-driven decision-making, have the potential to improve efficiency, enhance accuracy, and support proactive management of food safety and quality (Hooda et al., 2025; Plakantara and Karakitsiou, 2025). Despite these potential benefits, adoption of digital solutions in the dairy industry remains inconsistent, with many organizations relying on traditional methods or facing barriers to implementation (Shepherd et al., 2020; Plakantara and Karakitsiou, 2025). Understanding the specific needs, challenges, and opportunities for digital tools in this context is critical for designing solutions that are practical and effective. The objective of this study was to assess dairy industry needs for digital solutions that support food safety and quality management, using interviews to gather insights directly from food safety and quality professionals in the dairy sector.

To achieve the objective of our study, we conducted interviews as part of the National Science Foundation Regional I-Corps Program to evaluate potential users' needs and interest in digital tools. Participants were recruited using a combination of purposive and snowball sampling approaches. We purposively targeted food safety and quality managers and scientists from larger US dairy companies (>1,000 employees), who were considered likely early adopters of digital tools. However, the final sample also included participants from smaller companies (500–1,000 and <500 employees), allowing us to obtain perspectives from across a range of company sizes. Initial participants were identified through established professional relationships with the Cornell Food Safety and Milk Quality Improvement Program. Additional participants were recruited via snowball sampling, in which interviewees referred other qualified professionals who met the study criteria. To protect participant confidentiality, all interview responses were presented anonymously, whether quoted directly (marked by quotation marks), paraphrased, or summarized.

Semi-structured interviews were conducted by the first author (JS) in July and August of 2023 via teleconference. The interviews aimed to test 3 key hypotheses: (1) food safety and quality managers and scientists have a need for digital tools to support their work, including meeting regulatory compliance requirements and managing crisis situations such as product recalls; (2) they are actively seeking new digital solutions to improve their processes; and (3) they show interest in predictive digital tools capable of anticipating issues and informing decision-making. A total of 23 participants from 18 companies (effectively covering the entire US) were interviewed. Detailed notes documenting participants' responses were typed by the interviewer in real time throughout each interview and served as the primary data for analysis. The interview protocol covered participants' daily responsibilities, key challenges, and the digital tools they use. Participants who reported using digital tools were asked to describe specific instances of use and any plans for improvement. The interview prompts and questions included the following:

1. Please describe your day-to-day responsibilities.
2. What are the major challenges you face in your role?
3. Can you provide examples of digital tools you use in your work?
4. (For participants using digital tools) Please describe a specific instance in which you used digital tools in your job.
5. (For participants using digital tools) Are there plans to expand the digital tools you currently use? If so, how?

Qualitative analysis was conducted on detailed notes from the interviews to identify recurring themes and insights. We applied Braun and Clarke's approach to reflexive thematic analysis, which involved 6 phases: (1) familiarizing ourselves with the data, (2) generating initial codes, (3) constructing themes, (4) reviewing potential themes, (5) defining and naming themes, and (6) producing the report (Byrne, 2022). Semantic coding was used to capture the explicit, surface-level content of participants' responses, such as categorizing the digital tools currently in use by their specific functions. In contrast, inductive coding was used to identify more complex or nuanced insights that emerged from the data, including how regulatory pressures motivate companies to advance digitalization and how return on investment (**ROI**) considerations shape their decision-making. The initial coding was conducted in MAXQDA Analytics Pro (24.10.0, VERBI GmbH, Berlin, Germany). We examined the full set of codes, identified patterns across participants' responses, and aggregated related codes into broader themes that captured the dominant ideas emerging from the interviews.

The participants in our interviews held diverse roles and responsibilities across organizations of varying sizes. Table 1 provides an overview of these participants, organized by company size and professional position.

**Table 1:** Company sizes and professional roles of interview participants

Company size (no. of employees)	Professional role	n
10,000+	Vice president of quality assurance	1
	Director of food safety and quality	4
	Manager of food safety, quality, and laboratory service	2
	Food safety microbiologist	1
5,000–10,000	Manager of quality assurance, quality system, and technology	2
1,000–5,000	Vice president of quality, food safety, and regulatory	1
	Director of food safety and regulatory compliance	1
	Manager of food safety and laboratory	3
	Food safety microbiologist	2
	Data analyst—compliance	1
500–1,000	Director of quality, innovation, and tech services	2
	Senior microbiologist	1
	Senior product development and research scientist	1
<500	Vice president of research and product development	1
Total no. of interviews		23

Through thematic analysis, we systematically grouped individual codes into subthemes and synthesized these into 5 overarching themes that capture the current state, drivers, and needs for digitalization in the dairy industry (Figure 1). The following paragraphs examine each theme in greater depth.

**Figure 1:**
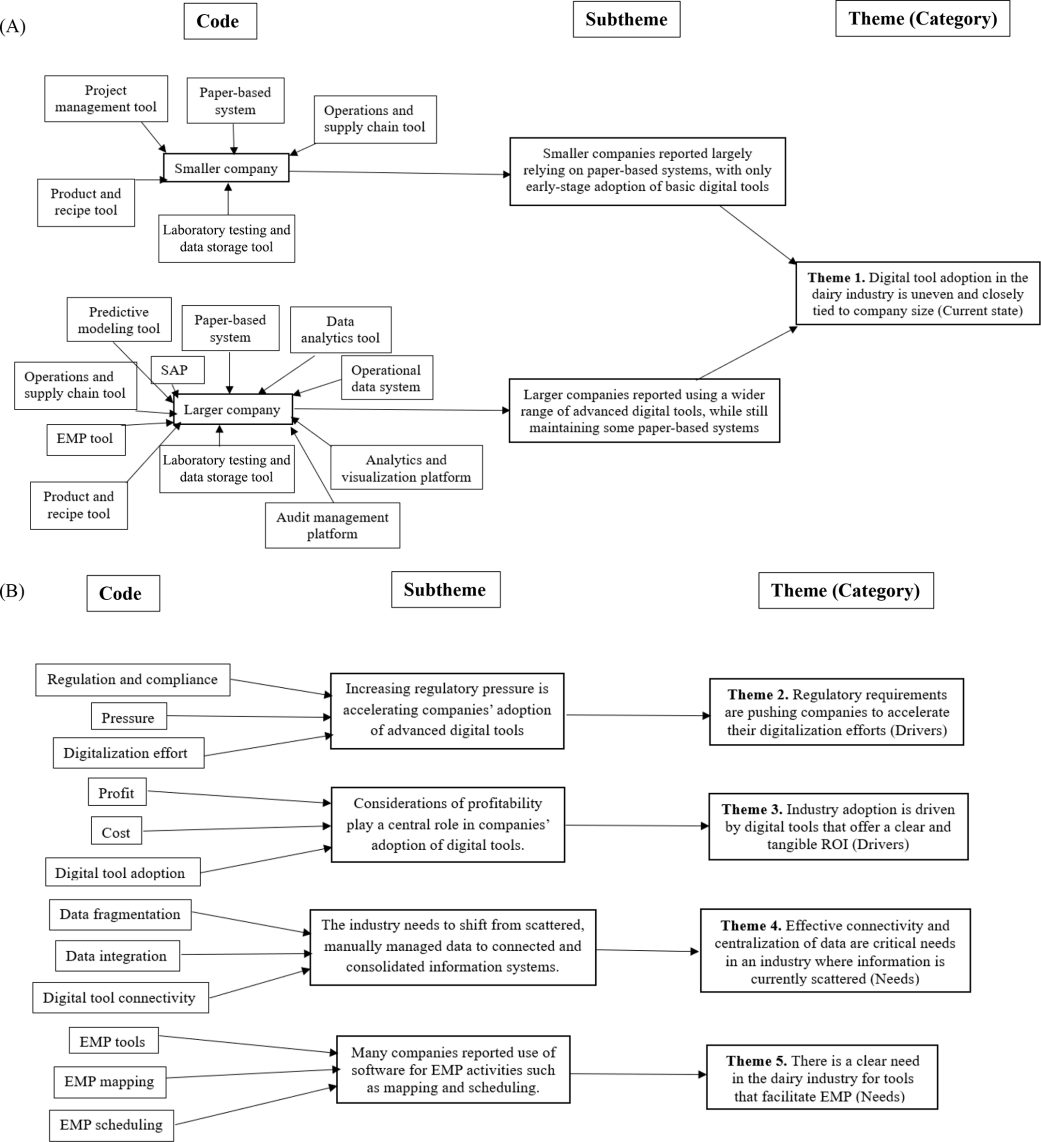
Organization of interview data from 23 dairy food safety and quality professionals on digital tool needs, showing how codes (i.e., a short label that captures the meaning of a specific segment of data) were aggregated into subthemes (i.e., a category that organizes multiple related codes into a meaningful pattern within a larger theme) and synthesized into 5 broad themes. Panel A depicts theme 1, and panel B depicts themes 2 to 5.

The first theme is that digital tool adoption in the dairy industry is uneven and closely tied to company size. Although larger dairy companies were more likely to adopt digital tools, regardless of size, companies reported continued heavy reliance on paper-based systems, and often Microsoft Excel spreadsheets, with 10 of the 18 interviewed firms reporting ongoing dependence on paper-based systems. This was particularly evident among smaller companies (i.e., 3 of the 4 interviewed smaller companies with <1,000 employees), which responded that they either relied almost entirely on paper-based systems or had only just begun using simple digital tools to manage projects, laboratory data, specifications, or standard operating procedures. Larger firms (>1,000 employees) had indicated adoption of a broader range of digital tools, though some paper-based systems persisted. Some tools, such as those for daily data tracking and trending, were reportedly used routinely, whereas other tools supported more advanced functions. One company subscribed to a commercial predictive modeling platform and hired an in-house analytics specialist to develop internal models, and several others used supplier management software to track ingredients. Other larger firms used ComBase (https://combase.errc.ars.usda.gov) for predictive modeling, laboratory information management systems (LIMS) for laboratory data management, and commercial software for environmental monitoring, as well as collaborated with external software companies to build supply chain management tools, creating multilayered digital ecosystems that reflect a more advanced yet still evolving stage of digital transformation. Notably, larger companies also reported leveraging artificial intelligence (**AI**) more actively, using structured and integrated data to generate predictive insights and optimize food safety and quality operations, whereas smaller firms reported limited exposure to AI tools at this stage. These findings are consistent with previous studies in other industries (Clemente-Almendros et al., 2024) and with research showing that small and medium-sized food companies face challenges in adopting integrated digital and innovative technologies due to limited financial and technical capacity (Gupta et al., 2025).

The second theme is that regulatory requirements are pushing companies to accelerate their digitalization efforts. Interview responses indicate that increasingly stringent food safety and traceability regulations are motivating companies to adopt more advanced digital systems. Managers and vice presidents from several larger companies, interviewed in July and August of 2023, highlighted that the US Food and Drug Administration's newly finalized traceability rule (issued in November 2022 with a 2026 compliance deadline at the time) was already influencing their operations. A manager from a company with over 5,000 employees explained that the rules' detailed requirements and short compliance timeline placed significant stress on the information technology department and required a 4- to 5-year digitalization project, pushing the company toward more advanced digital systems. Another manager described how their company updated software to meet traceability requirements, using tools that aggregate data from multiple sources, automate daily reporting, and ensure compliance for regulated products. A third manager emphasized the company's goal to bridge the gap between existing food safety systems and government standards, demonstrating a commitment to staying at the forefront of digital technology. Overall, discussions highlighted that regulatory pressures are prompting companies to modernize digital systems, integrate data across operations, and proactively align food safety practices with government standards. These findings are supported by prior work showing that regulatory pressures encourage food companies to accelerate digital adoption, particularly in supply chain management and traceability systems (Coltman, 2025).

The third theme is that industry adoption is driven by digital tools that offer a clear and tangible ROI. Our interviews indicated that dairy companies prioritize digital tools that directly support profitability, particularly in low-margin segments such as fluid milk products. Managers noted that budgets for departments such as food safety are often limited, making investments in expensive software difficult to justify unless the tool clearly contributes to revenue generation or cost savings. For example, one company reported relying primarily on Excel and mostly manual tracking systems, explaining that they had not adopted commercial supplier management software because it was too costly relative to the perceived return. This highlights that financial considerations strongly influence which digital tools are adopted, with preference given to solutions that demonstrate a measurable impact on efficiency, compliance, or profitability. This finding is consistent with a previous study indicating that food and beverage companies often adopt digital tools when they offer a clear and tangible ROI, such as cost savings, improved efficiency, and data-driven decision-making (Morrison, 2025).

The fourth theme is that effective connectivity and centralization of data are critical needs in an industry where information is currently scattered. Several food safety scientists and managers highlighted the challenges of working with data that were not stored in a single location, often requiring manual entry, data transfers from equipment to databases, and reliance on multiple disconnected systems for data storage. These processes increase the risk of human errors and inefficiency. Companies stressed the need to connect data from different sources, including sales, specifications, and laboratory systems, and to ensure connectivity across all digital tools. Many emphasized the value of a single digital solution that serves as a “single source of truth,”—a phrase used by a Food Safety and Quality Director from a larger dairy company (10,000+ employees), with similar sentiments expressed by others—to support streamlined workflows, accurate reporting, and better decision-making. These findings suggest, in our interpretation, that centralized and integrated data systems will also enable AI applications, as AI relies on connected, high-quality datasets to provide predictive analytics, anomaly detection, and operational optimization.

The fifth them is that there is a clear need in the dairy industry for tools that facilitate EMP. Of the 18 companies surveyed, 7 (all with >1,000 employees) reported using commercially available environmental monitoring program (**EMP**) software, noting its value for scheduling, mapping, and data management. They explained that these tools enable companies to manage environmental sampling data, track and trend results, and make informed decisions, including identifying high-risk sites and determining optimal sampling locations. A director of food safety and regulatory compliance from a larger company emphasized the pressure of maintaining compliance with the zero-tolerance policy for *Listeria monocytogenes* in ready-to-eat foods, noting that detection of *Listeria* in the processing environment necessitates immediate corrective actions. He noted that EMP software supports these efforts by enhancing mapping, centralizing data, and facilitating proactive decision-making, helping companies to uphold rigorous safety standards while efficiently managing resources.

Overall, our analysis highlights the main factors influencing digital adoption in the dairy industry. The interviews conducted suggest that implementation is uneven and closely linked to company size, with larger firms being more advanced in digitalization, whereas smaller companies remain in the early stages. Regulatory demands, including US Food and Drug Administration traceability rules and zero-tolerance food safety standards, are driving investment in tools that ensure compliance. Financial considerations also shape adoption, with priority given to solutions that offer clear returns, particularly in low-margin segments. Centralized and connected data systems are essential to address fragmented, manual processes and enable timely, accurate decision-making. Additionally, there is a growing reliance on EMP software to map, monitor, and analyze environmental data for proactive food safety management. Many of these findings are also relevant to the potential adoption of AI in the industry.
